# On Analyzing How the Th1/Th2 Phenotype of an Immune Response Is Determined: Classical Observations Must Not Be Ignored

**DOI:** 10.3389/fimmu.2019.01234

**Published:** 2019-06-05

**Authors:** Peter Bretscher

**Affiliations:** Department of Biochemistry, Microbiology and Immunology, University of Saskatchewan, Saskatoon, SK, Canada

**Keywords:** Th1/Th2 balance, pathogens susceptible only to Th1 immuniy, immunotherapy of cancer, immunotherapy of autoimmunity, B cells as APC, antigen dose

## Abstract

How an antigen interacts differently with lymphocytes and other cells of the immune system, to result in the generation of distinct classes of immunity, is one of the most basic questions of immune regulation. Understanding the nature of these “decision criteria” is central to developing effective medical interventions. Clinical observations lead to the recognition that much disease is due to an inappropriate class of immunity being generated, inappropriate because damaging, as in autoimmunity and allergies, or inappropriate because ineffective, against pathogens and cancer. I argue that the prevalent, contemporary conceptual frameworks, employed to analyze the nature of the decision criterion controlling the Th1/Th2 phenotype of the immune response, are implausible, as they ignore pertinent, classical observations. I outline reasons for favoring a different framework, that takes these observations into account, and explore its pertinence to the design of strategies of medical intervention.

## Understanding Immune Class Regulation Is Critical for Diverse Medical Interventions

Vaccination against pathogens contained by antibodies is immunology's greatest success. However, some pathogens, such as *Mycobacterium tuberculosis* (1, 2) and HIV-1 (3), are best constrained by cell-mediated immunity (CMI). Effective vaccination must guarantee CMI upon natural infection (4). In some cases of autoreactivity, pathological autoimmunity occurs only if autoreactive Th1 cells and CTL are generated, whereas similar autoreactive Th2 cells are non-pathological, as in autoimmune diabetes (5). Most cancers are progressive if the cytotoxic T lymphocyte (CTL) response is too weak, either because the cancer is insufficiently immunogenic (6), or because a substantial Th2 or other component of the response down-regulates the generation of CTL (7, 8). Allergic and non-allergic individuals, living in the same geographical area, predominantly produce IgE/ IgG_1_ and IgA/IgG_4_ antibodies against the allergen (9–12). Thus, understanding immune class regulation should allow the design of strategies to control the class generated, with medical implications. I end this article with a discussion of such strategies.

## Simplifications

People can produce IgM, IgA, and IgE classes of antibody and antibody belonging to the IgG_1_-IgG_4_ subclasses. Moreover, these classes/subclasses are differentially regulated. The two main classes of CMI, namely delayed type hypersensitivity (DTH) and CTLs, can be generated exclusive of antibody production (13).

We need, to get optimal insights into immune class regulation, to systematically examine how different variables of immunization affect the generation of *all* these classes/subclasses of immunity. No studies reflect such a program. How can we then make headway?

Older studies, initiated in the 1950s, characterized some variables of immunization that differentially affect the generation of DTH and IgG antibody production (14–16). Exclusive DTH corresponds in mice to the exclusive generation of Th1 cells (4). IgG_1_ antibody production exclusive of DTH corresponds to the generation of Th2 cells (4). Mixed Th1/Th2 responses have intermediate correlates: poor DTH and, in the mouse, IgG_2a_ and IgG_1_ antibody (17) and, in people, IgG_1_ and IgG_2_ antibody production (18). This context allows insights into how Th1 and Th2 cells are differentially generated. This insight, though not comprehensive, is sufficiently substantial to allow the design of strategies of medical intervention in diverse areas of medicine (19, 20), as discussed below.

## What Controls the Differential Generation of Th1 and Th2 Cells?

Various studies led to the Cytokine Milieu and the pathogen-associated molecular pattern (PAMP) Hypotheses, the most popular, contemporary models.

The *in vitro* presence of IL-12 and/or IFN-γ favors the generation of Th1 cells (21, 22) and of IL-4 the generation of Th2 cells (23). *In-vivo* observations in the mouse model of cutaneous leishmaniasis leave no doubt that IFN-γ and IL-4 are required for the generation of Th1 (24) and Th2 (25) responses. Many believe that the milieu must contain IL-12 and/or IFN-γ to generate Th1 and IL-4 to generate Th2 cells.

The PAMP Hypothesis reflects Janeway's legacy. He proposed that the activation of CD4 T cells requires PAMPs to stimulate APC to express costimulatory (CoS) molecules required for CD4 T cell activation (26–28). Many propose that PAMPs also determine the subset of CD4 T cell generated (29). Matzinger's Danger Model states that PAMPS/alarmins are required for the APC to express the requisite CoS molecules (30–32). Matzinger suggests a combination of PAMPs, alarmins and local tissue-factors determine the subset of CD4 T cell generated (31, 32).

## Variables of Immunization Generally Affecting the Th1/Th2 Phenotype of the Response

These variables should be accounted for by any decision criterion purporting to explain how the Th1/Th2 phenotype of activated Th cells is determined. To set the scene, I describe three such variables, first characterized 40–60 years ago, and subsequently confirmed many times. These variables appear to be generally pertinent.

**Figure 1:**
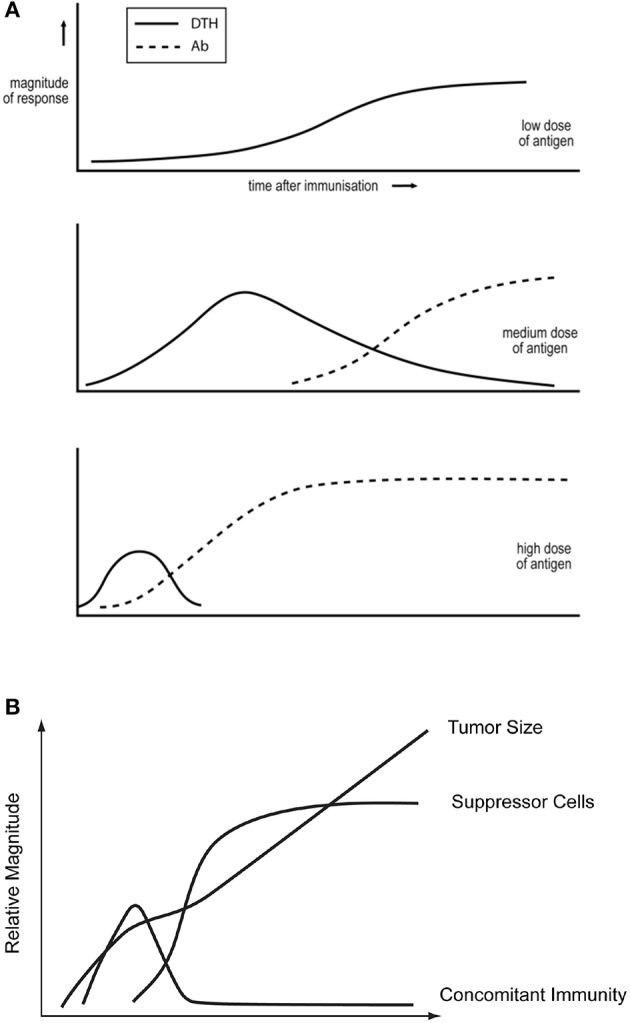
**(A)** Dependence of the DTH/IgG antibody nature of the immune response on antigen dose and time after immunization. Based on observations of reference Parish (15) and taken from reference Bretscher (33). (**B)** The kinetics of tumor growth, of the generation of concomitant immunity and of T cells able to suppress concomitant immunity, following the inoculation of a lethal dose of tumor cells. Based upon the studies of North described in North (8).

Salvin showed that antigen dose, and time after immunization, affect the DTH/IgG antibody nature of the ensuing responses (14), see Figure 1A. Moderate doses first induce DTH and, with time, the response evolves toward an IgG mode, with minimal expression of DTH. Larger doses lead to faster responses, and the DTH phase may be eclipsed. Smaller doses decrease the tempo. There may only be a DTH phase. These generalizations are supported by many studies. For example, they are true for SRBC (16) and leishmania parasites (4) and mycobacteria (34) in mice, for mycobacteria in cattle (35), and SIV in macaques (36).

Raffel argued, in 1968, on the basis of observation, that minimally foreign antigens are only able to generate DTH responses (37). Antibody against such antigens could be generated upon immunization with a conjugate of this antigen with a more foreign antigen. Making an antigen more foreign, by coupling a foreign antigen to it, modulates the response against the antigen from a Th1 toward a Th2 mode (38).

## The PAMP and Cytokine Milieu Hypotheses Cannot Account for These Variables

Consider the evolution of the immune response against a foreign, vertebrate, PAMP-free antigen, such as sheep red blood cells in mice, from a cell-mediated to humoral mode (16). The PAMP Hypothesis cannot explain this evolution, as the antigen expresses no PAMPs. The similar evolution of the response induced by PAMP-expressing entities, such as *L major* parasites (4, 17), is also problematic, as the PAMPS do not change with time. Similarly, these observations are not readily accommodated with the Cytokine Milieu Hypothesis. The other two generalizations, on antigen dose and the nature of the antigen, are also not readily understood in terms of either of these hypotheses.

Most of the observations that led to The Cytokine Milieu Hypothesis can be accounted for by a different hypothesis, The Cytokine Implementation Hypothesis, as outlined elsewhere (19). Briefly, it is generally acknowledged that cytokines, made by CD4 T cells belonging to one subset, favor the further generation of CD4 T cells belonging to this subset, and disfavor the generation of CD4 T cells belonging to opposing subsets. Thus, the immune response evolves to become more “coherent.” This proposal leaves open the question of what determines the initial, preferential generation of CD4 T cells of one subset over those of another subset? We address this question below.

We have tested the Cytokine Implementation Hypothesis in the case of IL-4 and Th2 cells. The IL-4 required to generate Th2 cells is made by CD4 T cells themselves (39), rather than by other cells in their environment.

## Immune Deviation

Two phenomena, discovered in the 1960s are important in understanding immune class regulation. Immunization leading to strong antibody production renders animals unresponsive for the induction of DTH, i.e., to “humoral immune deviation” (40). Animals immunized to generate sustained DTH can become unresponsive for the induction of antibody, a state of “cell-mediated immune deviation” (15). These states, I argue later, are critical for strategies of immunological intervention.

## The Threshold Hypothesis

I proposed this hypothesis, in 1974, to explain how the “Th1/Th2 phenotype” of a response is determined (41). The hypothesis accounts for the three sets of quantitative observations outlined above, on variables of immunization that affect the DTH/antibody nature of the immune response. Predictions of this hypothesis have been successfully tested in diverse ways, as recently reviewed (33). However, the hypothesis has had little impact. I believe this hypothesis is important both for understanding immune regulation and for developing strategies of medical intervention. I hope this article will foster both agendas.

Any decision criterion, as to how different CD4 T cell subsets are differentially generated, is inevitably cast in terms of what is envisioned to be required to activate CD4 T cells. Most think a single CD4 T cell can be activated by antigen under dangerous circumstances (26–32). In contrast, I believe that CD4 T cell activation requires CD4 T cell cooperation (41, 42), as recently reviewed (33). I suggest this proposition is essential for a realistic discussion of the nature of the decision criterion controlling the Th1/Th2 phenotype of an immune response. I imagine most immunologists have been disinclined to seriously consider The Threshold Hypothesis, as they were not ready to seriously entertain the idea that CD4 T cell activation requires antigen-mediated interactions between CD4 T cells. I have discussed elsewhere the reasons (42–44) and substantial evidence (38, 45–49) for a particular model for the activation of CD4 T cells. It is sufficient, for our present purposes, to consider that CD4 T cell activation requires antigen-mediated CD4 T cell cooperation mediated by an antigen-specific B cell acting as an APC.

The Threshold Hypothesis states that tentative and robust cooperation between CD4 T cells results, respectively, in the generation of Th1 and Th2 cells. This assessment of the “strength of cooperation” likely reflects the frequency of responder CD4 T cells forming synapses with B cells, previously activated by CD4 T cells. This hypothesis accounts for the three quantitative generalizations outlined above. (i) There are very few CD4 T cells specific for peripheral self-antigens (43), and few CD4 T cells specific for minimally foreign antigens. Even in the presence of amounts of antigen leading to efficient antigen presentation by B cells, CD4 T cell cooperation will only be tentative, leading to the exclusive generation of Th1 cells. (ii) There are more CD4 T cells specific for more foreign antigens. In the presence of amounts of antigen leading to efficient B cell presentation, there will be robust CD4 T cell cooperation and the generation of Th2 cells. A sufficiently lower dose, leading to less efficient B cell presentation, will support only tentative co-operation, and the generation of Th1 cells, thus accounting for Salvin's observations on antigen dose (14). (iii) When a foreign antigen impacts the immune system, it initially causes CD4 T cells to multiply. Thus, so long as the level of antigen is sustained, the degree of CD4 T cell cooperation intensifies, so the immune response can evolve from a Th1 toward a Th2 mode (41), in accordance with observation (Figure 1A). My purpose here is not to describe and justify this model in detail, as this has been done recently (33). Rather, I want to indicate its plausibility and explore its potential for designing strategies of medical intervention. However, one validated prediction can be exploited to modulate immune responses: a partial depletion of CD4 T cells, around the time of antigen impact, can switch a response from what would have been a Th2 to a Th1 mode, all other variables being kept constant. This prediction has been verified in many systems (38, 39, 50–52), and is paradoxical in the context of the PAMP and Cytokine Milieu Hypotheses (33). Some cellular/molecular details of how the threshold mechanism is realized have been experimentally delineated. It appears that the strength of B cell-mediated CD4 T cell cooperation is assessed via the CD28/B7 costimulatory systems (38, 39, 49, 51).

Chronic and tentative CD4 T cell cooperation, due to the sustained presence of antigen, can result in a build-up of Th1 and associated antigen-specific CD8 T cells that inhibit the generation of Th2 cells (15, 53–55). This state of cell-mediated immune deviation can be exploited for medical purposes (19), as discussed below.

## The Threshold Mechanism and Strategies of Medical Intervention

Those believing in the threshold mechanism's plausibility will consider different approaches to achieving some medical goals than those who do not. I conclude by briefly examining various observations and strategies of intervention in the context of this mechanism.

### Infectious Diseases

BALB/c mice, infected with 10^6^ *L major* parasites, rapidly generate a sustained Th2 response and are consequently “susceptible.” These “susceptible” mice generate a stable Th1 response upon infection with 300 parasites, and consequently resist this infection. Such mice acquire with time a Th1 imprint and so make a Th1 response and resist a challenge of 10^6^ parasites (4). This is the basis of the low dose vaccination strategy against pathogens uniquely susceptible to cell-mediated attack (56), a strategy likely pertinent against HIV (13) and tuberculosis (35, 57).

In addition, normal, susceptible BALB/c mice can also be made resistant to 10^6^ parasites by a partial depletion of CD4 T cells around the time of infection, associated with a modulation of the response from a Th2 to Th1 mode (52). This finding is anticipated on the Threshold Hypothesis but paradoxical in the context of the PAMP and Cytokine Milieu Hypotheses.

An on-going, anti-pathogen Th1/Th2 response of patients with visceral leishmaniasis is modulated to a Th1 mode on reducing the parasite load by administering anti-parasite drugs, constituting effective treatment (18). Mice with chronic cutaneous leishmaniasis, making a Th1/Th2 response against the pathogen, can be cured by partial depletion of CD4 T cells, resulting in a Th1 response (17). It therefore appears that antigen dose and CD4 T cell number not only determine the Th1/Th2 phenotype of primary but of on-going immune responses. This inference has obvious implications for treatment (19, 20), as discussed below.

A major impediment to universally effective vaccination is the genetic diversity of the population to be immunized. We have explored a means by which this impediment might be overcome, as described elsewhere (53). It is interesting from the perspective of the low dose vaccination strategy that BCG vaccination of cattle against tuberculosis has, for the most part, been ineffective. Buddle et al. appear to have shown that immunization with a million fold fewer BCG, than commonly explored, results in remarkable protection against an otherwise lethal challenge of *M. bovis* (35).

### Autoimmunity

The non-obese diabetic (NOD) mouse strain provides a model of human autoimmune diabetes. Pathological autoimmunity is due to Th1 cells and associated CD8 CTL, and Th2 responses are not pathological (5). One likely way of preventing disease would be to immunize very young, healthy mice, with substantial doses of β-islet antigens coupled to a very foreign protein, in a manner that biases responses to β-islet antigens into a Th2 mode (19, 20).

### Cancer Immunology

Most cancers are susceptible to CTL (6). North showed in the 1980s/1990s that mice, given a lethal dose of a transplantable tumor, first generate protective CTL, responsible for “concomitant immunity.” The sustained presence of these CTL is inhibited by the subsequent generation of “suppressor” CD4 T cells (8, 58, 59), see Figure 1B, and so to tumor progression. North made similar findings with diverse tumors. The similarity of Figures 1A,B led us to propose the Th2-skewing Hypothesis of Tumor Escape. We employed two tumors studied by North to successfully test this idea (7). Three older findings in the field of tumor immunology make eminent sense in terms of this hypothesis and of the threshold mechanism.

Firstly, mice given a lethal tumor challenge reject a second lethal challenge given about 9 days after the first, reflecting the presence of protective cells able to contain the smaller, second but “lethal” challenge (58). The generation of such “concomitant immunity” was discovered to be a rather general phenomenon in the 1950s (60), leading to the conclusion that most experimental tumors are immunogenic. Secondly, it had been discovered in the 1950s that operating out, or excising, a tumor, about 9 days after implantation of a lethal challenge, at a time now recognized to be a time when substantial, Th1-associated concomitant immunity has been generated, led in time to resistance against the tumor (61). We suggest that tumor excision greatly reduces the antigen load, precluding progression of the anti-tumor Th1 response toward a Th1/Th2 mode. We envisage the mechanism underlying “excision-priming” is similar to that underlying low dose vaccination (7). Lastly, human cancers are treated by their partial removal through surgery and by chemotherapy. Removing most of the cancer may sometimes modulate the anti-cancer response from a mixed Th1/Th2 toward a Th1 mode by reducing the antigen load, as occurs in the drug-dependent treatment of visceral leishmaniasis, see above. North has shown that one form of experimental chemotherapy, namely the administration of cyclophosphamide, is effective not (only) because it kills tumor cells, but by killing dividing CD4 T cells of a mouse given a lethal tumor challenge (62). A similar dose of cyclophosphamide, similarly administered at the time of antigen administration, switches the immune response of mice against SRBC from a humoral to a cell-mediated mode (63). We suggest the efficacy of this treatment is due to the fact that CD4 T cell depletion switches the anti-tumor response from a Th2 or Th1/Th2 toward a Th1 mode, as anticipated on the threshold mechanism. These considerations could lead to a personalized form of cancer treatment for those cancers where progression is associated with a mixed Th1/Th2 response (19, 20).

## Conclusions

The Threshold Hypothesis accounts for diverse observations on the variables of immunization known to affect the Th1/Th2 phenotype of the ensuing response. It is also supported by tests of its critical prediction, that partial depletion of CD4 T cells, around the time of antigen impact, all other variables being kept constant, modulates a Th2 or mixed Th1/Th2 response to a Th1 mode. This finding is paradoxical for the PAMP and Cytokine Milieu Hypotheses. The envisaged threshold mechanism provides a framework for explaining the efficacy of, and for designing, strategies of immunological intervention in different areas of medicine related to the immune system.

## Response to Reviews

The first two reviews of the first draft of this manuscript were unenthusiastic. The main criticism by both reviewers was that the threshold mechanism was inadequate to explain many findings made in the last 45 years since it was proposed. However, no observations from these voluminous findings were cited by the reviewers as being against the threshold mechanism. I argue that the threshold mechanism accounts for three general variables of immunization that affect the Th1/Th2 phenotype of the response, and that these generalizations are difficult to square with the Cytokine Milieu and PAMP Theories. I could not tell whether the reviewers thought the generalizations invalid, the explanations invalid, or why this accounting is insignificant. I therefore made minimal changes to the text, except to indicate more details concerning the Cytokine Implementation Hypothesis, as requested, and shortening the paper, so I could respond here to the reviewers' comments. I thank one reviewer for endorsing the manuscript in view of these changes.

Both reviews made me realize that my understanding of how science progresses is different from the reviewers'. We do not reject The Clonal Selection Theory because it does not account for recent findings; rather, we embrace the evidence supporting this theory and employ it as a framework for further understanding. No theory is ever complete. The threshold mechanism is quantitative, and brings together quantitative aspects concerning the nature of the antigen, antigen dose and time after immunization. I consider this to be a unique feature for a hypothetical mechanism as to how the Th1/Th2 phenotype of an immune response is determined. This hypothesis brings what are likely new insights into how immune responses against tumors and infectious agents are regulated, as recognized by one of the reviewers in the context of L major infection. This reviewer acknowledged that the effects of CD4 T cell depletion, in making susceptible mice resistant to a challenge of 106 parasites, was uniquely explained by the threshold mechanism. The Threshold Hypothesis makes testable and tested predictions. In contrast, both the PAMP and Cytokine Milieu Hypotheses are rather open-ended; there is little rationale given for how one PAMP defines the Th1/Th2 nature of the response, and so empirical studies are the primary way forward; what controls the cytokine milieu is not specified. These frameworks are insufficiently defined to allow the ready testing of specific predictions or to readily base strategies of immunological intervention. I am grateful to a third and later reviewer for his/her constructive comments.

## Author Contributions

The author confirms being the sole contributor of this work and has approved it for publication.

### Conflict of Interest Statement

The author declares that the research was conducted in the absence of any commercial or financial relationships that could be construed as a potential conflict of interest.

## Abbreviations

APC: antigen presenting cells
CoS: costimulation/costimulatory
CMI: cell-mediated immunity
CTL: cytotoxic T lymphocytes
DTH: delayed-type hypersensitivity
PAMPs: pathogen-associated molecular patterns.
